# Supporting Clinical Identification of Children with Sensory Integration Challenges: A Decision Guide for Primary Care Providers

**DOI:** 10.3390/brainsci15111184

**Published:** 2025-10-31

**Authors:** Shelly J. Lane, Sarah A. Schoen, Roseann Schaaf, Anita Bundy, Zoe Mailloux, Susanne Smith Roley, Teresa A. May-Benson, L. Diane Parham

**Affiliations:** 1 Department of Occupational Therapy, College of Health and Human Sciences, Colorado State University, Fort Collins, CO 80523, USA; anita.bundy@colostate.edu; 2 Department of Occupational Therapy, Rocky Mountain University of Health Professions, Provo, UT 84606, USA; dr.schoenphdotr@gmail.com; 3 Department of Occupational Therapy, Jefferson College Rehabilitation Sciences, Thomas Jefferson University, Philadelphia, PA 19107, USA; roseann.schaaf@jefferson.edu (R.S.); zoe.mailloux@gmail.com (Z.M.); 4 Susanne M Smith, Inc., Aliso Viejo, CA 92656, USA; susanner3@gmail.com; 5 TMB Education, 2305 Springview Rd, Norristown, PA 19401, USA; tmb@tmbeducation.com; 6 Occupational Therapy Program, School of Medicine, University of New Mexico, Albuquerque, NM 87106, USA; ldianeparham@gmail.com

**Keywords:** access to care, children and youth, sensory integration, primary care practitioners, clinical decision-making

## Abstract

**Background**: Children and youth often have challenges processing and integrating sensory information. These increasingly common challenges and can significantly impact development, learning, behavior, well-being, and participation in everyday activities. Since children with sensory integration challenges, with or without other concerns, are likely to present first to their primary care provider (PCP), it is important that they have resources about sensory integration challenges and their impact on the child or the need to refer these children for further assessment and intervention. Our aim is to assist PCPs in their clinical decision-making. **Methods**: We conducted a narrative review of CINAHL, PsychInfo, and Medline with no date restrictions, using the structure Population (children/youth with sensory integration/processing disorder/dysfunction/difference)/Concept (screening or referral)/Context (screening from PCP to occupational therapy) to identify the pertinent literature, providing (1) a description and synthesis of a circumscribed body of research on sensory integrative challenges; (2) findings related to screening and referral to occupational therapy by PCPs; and (3) the need for development of a Sensory Integration (SI) Decision Guide to support PCP clinical decision-making. **Results**: Findings from the narrative literature review search were integrated with information from the author panel of experts to provide a description of sensory integration challenges. Few screening tools were addressed in the literature, and no guidelines were identified to support PCP decision-making regarding referral. A Sensory Integration Decision Guide was developed to fill this gap. **Conclusions**: The Sensory Integration Decision Guide provides primary care providers with a systematic process for detecting sensory integration challenges and referring to specialized occupational therapy services. Future studies to examine the practical application of the tool for its accuracy and usefulness in clinical decision-making and effectiveness for referral decisions are needed.

## 1. Introduction

Children and youth often struggle to process and integrate sensory information, which, if left unrecognized and untreated, can result in negative consequences in everyday activities, routines and roles. Often referred to as challenges in sensory integration or sensory processing, these interconnected terms describe the neural processes through which we organize sensory inputs from the body and the environment and use this information to adaptively interact with the world [1]. Applying the theory of Ayres Sensory Integration^®^ [2], these functions are foundational to everyday activities, and disruptions can have pervasive effects on behavioral and emotional regulation, motor performance, social interaction, self-care, and learning. While readers may encounter these and various other terms in the literature or the vernacular of families or professionals, to promote clarity and consistency, in this paper, we employ two terms: (1) sensory integration, encompassing sensory responsivity, sensory perception, and sensory–motor abilities, and (2) occupational therapy using Ayres Sensory Integration^®^ (ASI) to refer to the theoretical model for assessment and intervention. We present this framework to assist readers in understanding how sensory integration challenges may manifest in children and to assist in ensuring the provision of appropriate services/supports when present.

Intervention based on Ayres Sensory Integration^®^ is considered the gold-standard for individuals with sensory challenges. Grounded in neuroscience [1], this intervention has specific requirements for therapist qualifications and the processes of assessment and intervention designed to remediate underlying sensory issues that affect functional performance [3,4]. It is described in detail by Schaaf and Mailloux, 2015 [5].

Differences in how we process and integrate sensation impact many facets of life. Researchers have shown that children with sensory integration challenges experience behavioral difficulties that interfere with their ability to engage in social interaction, play with peers [6,7], sleep adequately [8,9], benefit from learning environments [10], and engage in other routine childhood activities [11]. In addition, evidence indicates that parenting stress [12] and strain [13] is high when a child has sensory integration challenges and that families often make accommodations for their child, limiting participation in family routines and community activities [14,15,16,17].

Sensory integration challenges manifest as difficulties in sensory perception, hypo- or hyper-responsivity, and engagement in sensory–motor activities. Differences in sensory perception occur in any sensory system and may impact motor abilities. Responsivity differences also occur across sensory systems and are reflected in behaviors ranging from withdrawal from or intense response to sensation to not recognizing sensory cues from people and things in the environment. Sensory–motor difficulties include poor postural control and coordination of eye movements and challenges with motor planning /execution of movements [18]. Functionally, these differences are seen as motor clumsiness, challenges with balance, and difficulty with age-appropriate motor skill attainment [19]. Ultimately, these sensory integration challenges interfere with children’s abilities to engage in everyday occupations such as self-care, play, leisure, and school activities. Parents report that such challenges are among the most difficult issues they face in managing their child’s behavior and engagement in everyday activities. Consequently, parents rate occupational therapy intervention as a top priority for remediating these challenges [20,21,22].

Sensory integration challenges are often comorbid with existing neurodevelopmental or mental health conditions such as autism [23,24] and attention deficit hyperactivity disorder [ADHD] (e.g., [25]), as well as some psychiatric disorders. Additionally, these challenges can manifest in children with no other condition [26,27,28]. The high prevalence of sensory integration challenges in children with (50–90%) and without (5–20%) other diagnoses [26,27,28,29,30,31,32,33,34,35,36,37,38] highlights the importance of accurate detection and intervention.

Since children with sensory integration challenges, with or without other concerns, are likely to present first to their primary care provider (PCP), it is important that they have resources about sensory integration challenges and their impact on the child or the need to refer these children for further assessment and intervention. Our aim is to assist PCPs in their clinical decision-making. Toward this end, we summarize foundational information to support an understanding of these challenges, as well as the literature on screening and referral for sensory integration challenges. The goal of this narrative review is to facilitate accurate detection of children with sensory integration challenges in need of referral to an occupational therapist specializing in Ayres Sensory Integration ^®^ assessment and intervention.

Thus, this study comprises (1) a description and synthesis of a circumscribed body of research on sensory integrative challenges; (2) findings related to screening and referral to occupational therapy by PCPs; and (3) the need for development of a Sensory Integration (SI) Decision Guide to support clinical decision-making in the process of identification and referral of children with or without sensory integrative challenges.

## 2. Materials and Methods

A narrative review is a flexible methodology for synthesizing information in a meaningful way for a specific purpose. Although narrative reviews are non-systematic and less rigorous than scoping or systematic reviews, this approach provides an appropriate structure for synthesizing and interpreting the selected research related to the aim of this study [39,40]. Additionally, studies by the authors, acknowledged authorities in sensory integration, are also included. Because this study is a review of the existing literature, ethics approval was not required.

Our review is focused on assisting PCPs to recognize and understand behaviors that reflect sensory integration challenges; identify screening tools that substantiate a link to sensory integration; and refer appropriately to occupational therapy. In synthesizing findings, we sought to identify supports that might exist to inform PCPs’ clinical decision-making. We provide information to help PCPs reframe the way they think about child behaviors by considering how challenges in processing and integrating sensation impact daily life functioning. Additionally, based on the existing literature, we assess the need for a decision-making guide to support PCPs’ clinical judgement.

We structured our search strategy using Population/Concept/Context (PCC) rather than a Population/Intervention/Comparison/Outcome (PICO) strategy. We determined this to be appropriate for our narrative review because we were not investigating a specific intervention or outcome. We searched CINAHL, PsychInfo, and Medline, all through EBSCO. Key terms could be found in title, abstract, key terms, or text. See Table 1 for a full list. Our search terms were purposefully broad to identify as many articles as possible. Population terms were designed to capture the myriad terms used to describe sensory integrative challenges; children with such challenges were our population of interest. We included “screening” as a Concept term to capture the process of using a screening tool that would shed light on strengths and concerns for the population of interest. Referral terms, also an aspect of Concept, were identified to capture referrals from PCPs and other health professionals. For Context, we were interested in screening and referral from PCPs and referral to occupational therapy. We searched each term separately and subsequently combined terms to meet our aims. As such, the following combinations were searched:

(1) All population terms + screening;
(2) All population terms +all referral terms;
(3) (All population terms + screening) + all primary care terms;
(4) (All population terms +all referral terms) + all primary care terms;
(5) (All population terms + all referral terms) + all occupational therapy terms;
(6) ((All population terms + all referral terms) + (all occupational therapy terms) + (all primary care terms)).

We limited the search to children and youth and excluded works not written in English. There were no date restrictions; we excluded materials that were potentially not peer-reviewed (e.g., dissertations, practice journals, newsletters); we did not systematically search the gray literature.

We did not use GenAI at any point in the preparation of this manuscript.

## 3. Results

The results of this study represent an integration of data from the narrative literature review search, augmented by data from the author panel of experts. We organized outcomes by the overall goal of this study to support PCPs in accurately identifying and referring children with sensory integration challenges. Relevant background information includes a rationale for the need, preliminary data on prevalence, a description of the condition and the appropriate intervention, existing data on screening and referral, and need for the development of an SI Decision Guide.

### 3.1. Understanding Sensory Integration

Expertise in understanding sensory integration processes and challenges historically lies within occupational therapy [2]. Occupational therapists have built on the original theory, developed assessment tools, and conducted studies to test the effectiveness of the intervention. While sensory integration challenges may appear across child healthcare disciplines, the literature demonstrates a preponderance of studies within occupational therapy [6,8,35,41,42,43,44,45,46,47,48,49,50,51,52].

Our results reinforce that sensory integration challenges can be associated with other developmental, behavioral, and emotional challenges in children with no diagnosis, as well as those with comorbid diagnoses. The extent of these challenges is evident in a range of clinical groups such as children with ASD [31], ADHD [25], Down’s syndrome [53,54], fetal alcohol spectrum disorders [55,56], pediatric acute-onset neuropsychiatric syndrome [47], and preterm infants [57,58]. Within this literature is the recognition of the importance of educating physicians and caregivers about both the symptoms and impacts of sensory integration challenges on these clinical groups [58,59]. Prevalence estimates tend to vary by clinical condition, with values as high as 80% in ASD [60], 40% in ADHD [43], and 87% in preterm infants [59].

The literature also reveals specific clusters of symptoms as well as their impact. Sensory integration challenges include difficulties in sensory–motor processing, somatosensory processing, and/or sensory modulation [57]; these negatively affect a range of childhood activities, including performance of basic academic or daily life activities [47,61,62]. Sleeman and colleagues [63] indicated that the diversity, intensity of, and independence in these activities were impacted.

ASI is often referred to as the ‘gold-standard’ intervention for sensory integration challenges; it is certainly the most well studied. Studies demonstrating the effectiveness of ASI appear across populations with a wide range of child conditions including those with and without comorbid diagnoses. ASI is deemed an evidence-based approach for children with autism [50,64,65] and has been shown to produce positive outcomes across multiple populations [41,52,64,66,67]. A growing body of literature indicates maintenance of gains 6 to 12 months beyond immediate completion of an intervention program [45], in some cases extending to several years after intervention [44].

In summary, this narrative review reinforces earlier statements indicating that sensory integrative challenges are widespread across children with and without other conditions and that there are identifiable clusters of symptoms that have a meaningful negative impact on daily lives. Occupational therapists are the professionals with the greatest expertise in remediating these challenges. ASI intervention has been successfully used to support development and participation in daily life activities with the potential for long-lasting positive effects.

### 3.2. Screening and Referral

Investigators reported the use of a range of tools to identify sensory integration challenges, most qualifying as assessment rather than as screeners. Two tools identified could potentially be used for screening by PCPs: the Motor Planning Maze Assessment is suggested as a screening tool for sensory-based motor challenges, while the STAR Institute for Sensory Processing Disorder Checklist screens for several aspects of sensory integration, including reactivity, discrimination, motor praxis, and impact on childhood occupations. We also identified a new tool that may serve as a screener to investigate the impact of sensory integration challenges on participation in infancy (Sensory Integration Infant Routines Questionnaire) [68,69].

While not screening tools, we found other assessments in our search; they are those typically used by occupational therapists to aid in the assessment of sensory integration challenges.

Two standardized caregiver or self-report measures, often used in research and clinical practice, also appeared in our search; these have been adopted by professionals other than occupational therapists for screening purposes: The Sensory Profile-2 Suite [70] and The Sensory Processing Measure-2 [71]. Both sets of tools include multiple forms, spanning the age range and contexts of home, school, and community. We suggest these measures are best included as part of a comprehensive occupational therapy evaluation of sensory integration functions, although PCPs may find them useful for identifying sensory challenges.

Five publications [72,73,74,75,76] addressed appropriate referrals to occupational therapy by potential PCP professionals. The professionals standing as PCPs in these studies included child psychiatrists [72], social workers, [75], psychiatric nurses [74], and nurse practitioners [76,77]. No investigators provided or referenced specific screening tools, although most offered caregiver questions. Most authors agreed that occupational therapists have the greatest expertise in sensory integration and suggested the benefit of referral to occupational therapy.

### 3.3. The Need for a Sensory Integration Decision Guide

We were unable to find a decision guide to support PCP clinical decision-making for sensory integration challenges. Authors and investigators in professions that are front-line [72,73,74,75,76,77] indicated that practitioners in their profession need basic knowledge of sensory integration theory and practice. When faced with the task of identifying children who could benefit from occupational therapy, investigators suggested red-flag behaviors that could be used to inform referral. Examples include showing unusual responses to sensation, reflected in over- or under-responsivity to everyday sensation, challenges with sensory discrimination that may impact handwriting, gross or fine motor skill, balance, or postural control. Questions offered by Walbam (2014) [75] could facilitate information gathering from caregivers regarding potential sensory integration challenges:

- Is the child picky about certain tastes or textures when eating?
- Does the child have difficulty getting dressed or finding comfortable clothing?
- Has the child been sensitive to sounds?
- Does the child withdraw when there is noisy group play?
- Do sensory experiences like these interfere with social or academic functioning?

Importantly, authors recognized the overlap between, and/or co-existence of features of, sensory integration challenges and those of other conditions, such as attention deficit hyperactivity disorder, learning challenges, autism, and childhood affect regulation [72,73,74,75,76]. This overlap makes differential diagnosis challenging.

Thus, while existing recommendations and suggestions may be helpful, they fall short of a decision guide with a defined process and procedure for PCPs to use in identifying sensory integration challenges for the purpose of appropriate referrals. Decision guides or aids are common in many professions; they guide decision-making by providing practitioners with an evidence-based synthesis of relevant clinical indicators [78]. Rim and colleagues [79] indicated that such guides help practitioners make decisions about health care. We suggest that a decision guide could be useful for PCPs who see children with behavioral, emotional, and/or learning difficulties that may have a basis in sensory integration.

## 4. Discussion

Our narrative review supports the prevalence and impact of sensory integration challenges on a range of children. Coupling the identified literature with author knowledge, we document that these challenges often co-occur in children with a multitude of diagnoses, as well as in conjunction with concerns across behavioral [15], emotional [80], and developmental realms [81]. Thus, we sought information on screening and referral of children with sensory integration challenges to occupational therapy. We found few publications from only a handful of PCPs [72,73,74,75,76,77]. Those publications identify the need for knowledge of sensory integration challenges, and most recommended referral to occupational therapy. However, none provided a structured means to determine if sensory integration challenges contribute to caregiver concerns. This gap impacts the practice of health care professionals and, potentially, the welfare of affected children. Resources to support clinical decision-making for children and adults are common in the literature for other clinical conditions [79,82,83]. Such resources are essential tools to facilitate early identification and appropriate referrals.

**Figure 1:**
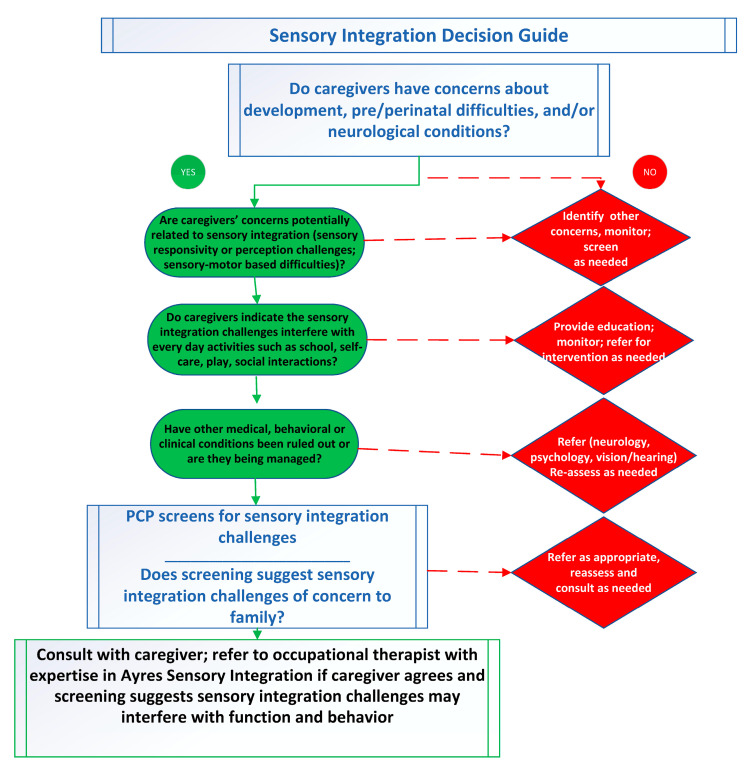
SI decision guide: grr.

In conducting this review, our aim was to identify the literature that could assist PCPs in their decision-making and potentially educate healthcare professions in the identification and referral of children with sensory integration challenges. Because of the prevalence of sensory integration challenges among children and youth and the absence of appropriate tools for making appropriate referrals for occupational therapy assessment and intervention, we created the Sensory Integration (SI) Decision Guide (Figure 1). As numerous authors [72,73,74,75,76,77] have indicated, PCPs require introductory knowledge of sensory integration theory to support clinical examination; this includes collecting information from caregivers as to the child’s functioning at home, school, and in the community. Children with sensory integration challenges often present with delays in self-care, dressing, and eating, as well as with motor coordination challenges, sensitivities, and challenging behaviors. These can be identified in conversation with the caregiver. Notably, there is high co-morbidity of these behaviors with autism, ADHD, and other neurodevelopmental or mental health conditions. While PCPs are in the best position to screen for these conditions, they should always look for the presence of co-occurring sensory integration challenges that may respond well to ASI and occupational therapy.

If the PCP identifies concerns about the child’s development, the decision guide can be used to help determine if these concerns have a sensory integrative basis or may be associated with some other neurodevelopmental condition. To achieve this, the SI Decision Guide includes guiding questions about childhood challenges and occupations that may be disrupted by sensory integration concerns. These questions indicate actions the PCP can take towards accurately identifying the child’s individualized needs. We present two pathways, one leading to referral to occupational therapy (green in Figure 1) and the other to screening for other conditions and monitoring or referral to other professionals (red in Figure 1). Regardless of whether a comorbid condition exists, if parental concerns or child history suggest challenges in sensory and motor abilities impacting daily life, the SI Decision Guide may be helpful in screening for sensory integration challenges. We offer two short case examples using the SI Decision Guide in the Supplementary Material.

While the literature we found in our review did not identify a wealth of screening tools, there are myriad checklists addressing sensory integrative challenges available on the web through occupational therapy clinics. Some examples of these checklists include those available from the Spiral Foundation (http://bit.ly/4pc0nY2, accessed on 23 October 2025, or https://www.thespiralfoundation.org/ash, accessed on 23 October 2025), and the STAR Institute (https://sensoryhealth.org/basic/symptoms-checklist, accessed on 23 October 2025). These non-standardized tools provide initial information as to the nature of the challenges but do not provide standard scores or scoring guidelines. They are a place to begin exploring sensory integration challenges and gain an initial understanding of reported concerns. Importantly, the most useful checklists include questions related to sensory perception, sensory responsivity, and sensory–motor abilities.

When available, PCPs should utilize standardized and validated tools. Both the Sensory Processing Measure-2 (SPM-2) [71] and the Sensory Profile-2 suite (SP-2) [70] deliver insight into sensory-integration-related challenges and provide standard scores. If screening or use of these standardized tools suggest sensory integration impairments, referral to an occupational therapist for a comprehensive assessment is a recommended next step.

When screening suggests sensory-integration-related challenges, we recommend collaborating with the family to determine needs, priorities, and preferences for their child as well as the contexts in which the child’s participation is compromised. If referral to occupational therapy is the next step, it is essential to select a therapist with appropriate training in Ayres Sensory Integration^®^ (ASI) intervention. Therapists trained in this approach have the advanced knowledge to ensure appropriate assessment and provision of intervention [4,84].

Occupational therapy using ASI is distinguished from other sensory-based interventions, such as “sensory diets,” or specific sensory strategies, such as a brushing protocol or listening to music on headphones [18]. Occupational therapy using ASI is the only evidence-based occupational therapy treatment for sensory integration challenges. There is good evidence for the efficacy of ASI with autistic children [5,48,64,65] and emerging evidence for its efficacy with children with a range of other conditions. These include, for instance, cerebral palsy and cortical visual impairment [85] and ADHD [32]. Emerging efficacy evidence is also available for children with no specific medical or behavioral diagnosis [41,42,45,46,52,86]. As an evidence-based intervention, some insurance carriers cover occupational therapy using ASI as a neurodevelopmental concern.

Children receiving ASI have realized numerous gains, including independence in daily activities, improved participation in social situations, and better regulated behavior [50,51,52,64,65]. Researchers have also reported that children experience improved receptive communication and fine and gross motor skills [41,42], functional regulation and participation in school [52], and gains in individually established family-centered goal-attainment-scaled goals [5,46].

We sought to identify tools and processes to assist PCPs to screen and refer children with potential sensory integration challenges for in-depth assessment and intervention. However, we found few existing tools. To reduce that gap, we developed the SI Decision Guide in the hope that families directed to the appropriate services for their child will experience much smoother daily life.

## 5. Conclusions and Future Directions

Prevalence of sensory integration challenges is high in children with and without other diagnosed conditions. However, as this review shows, there is little in the literature to support PCPs to identify signs of difficulties with sensory integration that impact everyday life. The SI Decision Guide developed in this research is the only tool of its kind supporting screening procedures to determine the need for assessment by a trained occupational therapist. When a sensory integration challenge is determined, the literature supports the use of an evidence-based occupational therapy intervention such as ASI.

The potential impact of this tool may be far-reaching. PCPs can feel more confident about appropriate referrals to an occupational therapist, and families can benefit from the support provided by their PCP in navigating their child’s treatment plan. If a trained occupational therapist confirms the presence of a sensory integration challenge and implements individualized intervention, children and families can expect the impact of such challenges to be minimized and daily life experiences maximized.

As a new tool, the SI Decision Guide requires empirical testing. Future studies are needed to evaluate its practical application. Such studies require both qualitative and quantitative data collection to examine the appropriateness, clarity, and completeness of referrals. Qualitative data from PCPs should focus on feedback related to clinical relevance in real-world use. Retrospective validation metrics from patient charts can address the sensitivity and specificity of the tool. Effectiveness regarding the SI Decision Guide’s impact on child outcomes in therapy will also be crucial [87]. Finally, it will be necessary to test the usefulness of the SI Decision Guide with other professionals who encounter children with sensory integration challenges, and are in a position to refer them to necessary services (e.g., teachers, social workers, nurse practitioners).

## 6. Limitations

This study has several limitations. First, it was inherently limited due to the choice of methodology. A narrative review is a structured-yet-flexible investigation of the literature, which, in this case, identified little support for PCPs for recognizing and referring children with potential sensory integration challenges. While this type of review inherently lacks the rigor of a systematic review, it was appropriate for this purpose. The study covered a broad overview of sensory integration challenges; nonetheless, what is presented is not an inclusive review of sensory integration. Second, by necessity, this narrative review included studies employing a wide variety of methodologies and provided an overall summary and interpretation of information related to sensory integration. Third, this narrative review selected only relevant sources regarding sensory integration that could be grouped meaningfully to support the rationale and procedures PCPs could use for identification and referral to occupational therapy services. As with many narrative reviews, some bias might exist due to the expert panel who reviewed and interpreted the literature. Lastly, this information is based on an understanding of the health care delivery in the United States and may not be applicable to other countries.

## Figures and Tables

**Table 1:** Search terms.

Population	(“sensory processing” or “sensory integration” or “sensory modulation” or “sensory-based motor”) AND (dysfunction or disorder or challenge or difficult *)	
Concept	(screening or “early detection” or “early diagnosis” or “early identification”)	(referral or “referral process” or “referral pathway” or “decision guide”)
Context	(“primary care provider” or “primary health care provider” or “primary healthcare provider”)	(“occupational therapy” or “occupational therapist” or “occupational therapists” or ot)

* this symbol indicates the term “difficult” could be present in form (e.g., difficulties, difficulty, etc.).

## Data Availability

No new data were created or analyzed in this study.

## Supplementary Materials

The following supporting information can be downloaded at: https://www.mdpi.com/article/10.3390/brainsci15111184/s1, Two short cases.
