# Supplementary material for: Supporting Clinical Identification of Children with Sensory Integration Challenges: A Decision Guide for Primary Care Providers
Source: Brain Sci. 2025 Oct 31;15(11):1184. doi: 10.3390/brainsci15111184 (PMC12650441; doi:10.3390/brainsci15111184)

Sensory Integration Decision Guide: Case 1

Case 1 Background

Capable 9 year old boy struggling with behavior at home and at school; sent home from school several times due to behaviors that staff could not control. He disliked the smells in the lunch room, did not tolerate people touching him, had difficulty focusing and was anxious, making work completion challenging.

Do caregivers have concerns about development, pre/perinatal difficulties, and/or neurological conditions?

Parents wer concerned about attention, impulsivity, and dysregulated and oppositional behavior

YES

NO

Monitor development; screen as needed

Are caregivers' concerns potentially related to sensory integration (sensory responsivity or perception challenges; postural or motor-based difficulties)?

Parents report extreme sensitivity to clothing texture, aversion to glue, obsession with clean hands, refusal to try new foods

Do the sensory integration challenges interfere with every day activities such as school, self-care, play, social interactions?

Provide education; monitor; refer for intervention as needed

Have other medical, behavioral or clinical conditions been ruled out or are they being managed?

He was diagnosed with ADHD and treated with medication but dysregulated behavior and sensory sensitivities continued to negatively impact his behavior at school

Refer (neurology, psychology, vision/hearing) Re-assess as needed

PCP screens for sensory integration challenges

Does screening suggest sensory integration challenges of concern to family?

Refer as appropriate, reassess and consult as needed

Refer to occupational therapist with expertise in Ayres Sensory Integration if screening suggests sensory integration challenges may interfere with function and behavior

Assessed by OT using Evaluation of Ayres Sensory Integration; scores within typical range. OT worked with caregivers and teachers to create supports (visual schedule, adaptations to daily routine, eating lunch outside, access to a quite space, designated workspace to support self-regulation. Recommendations made to consider a change in medications and seek mental health services. No OT services needed at present.

Sensory Integration Decision Guide: Case 2

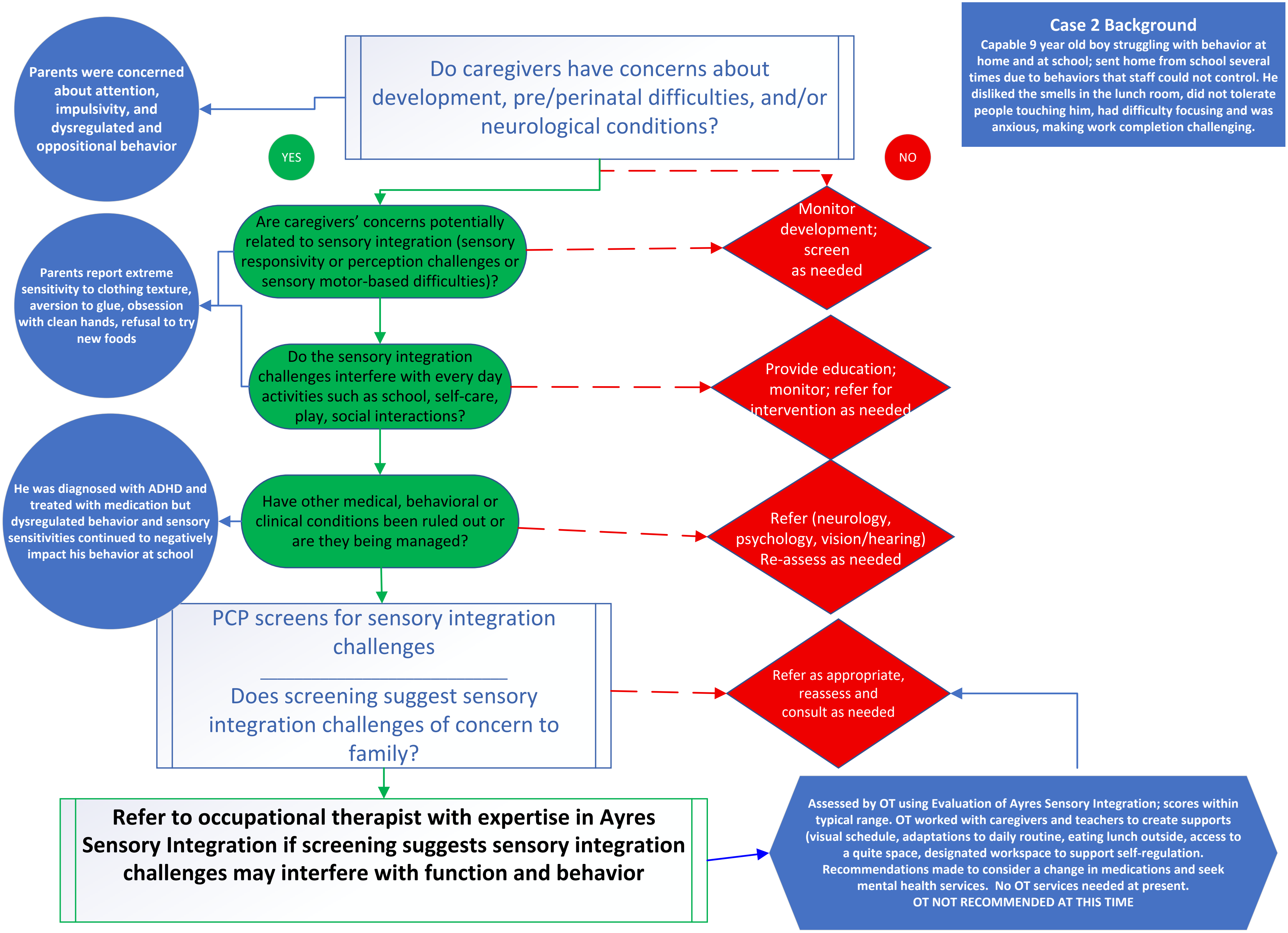

Supplement: Supplementary file 1 [file brainsci-15-01184-s001.zip › brainsci-3910855-supplementary.pdf]
